# Plant-Pollinator Coextinctions and the Loss of Plant Functional and Phylogenetic Diversity

**DOI:** 10.1371/journal.pone.0081242

**Published:** 2013-11-29

**Authors:** Marcos Costa Vieira, Marcus Vinicius Cianciaruso, Mário Almeida-Neto

**Affiliations:** 1 Programa de Pós-Graduação em Ecologia e Evolução, Universidade Federal de Goiás, Goiânia, Goiás, Brazil; 2 Departamento de Ecologia, Universidade Federal de Goiás, Goiânia, Goiás, Brazil; University of Marburg, Germany

## Abstract

Plant-pollinator coextinctions are likely to become more frequent as habitat alteration and climate change continue to threaten pollinators. The consequences of the resulting collapse of plant communities will depend partly on how quickly plant functional and phylogenetic diversity decline following pollinator extinctions. We investigated the functional and phylogenetic consequences of pollinator extinctions by simulating coextinctions in seven plant-pollinator networks coupled with independent data on plant phylogeny and functional traits. Declines in plant functional diversity were slower than expected under a scenario of random extinctions, while phylogenetic diversity often decreased faster than expected by chance. Our results show that plant functional diversity was relatively robust to plant-pollinator coextinctions, despite the underlying rapid loss of evolutionary history. Thus, our study suggests the possibility of uncoupled responses of functional and phylogenetic diversity to species coextinctions, highlighting the importance of considering both dimensions of biodiversity explicitly in ecological studies and when planning for the conservation of species and interactions.

## Introduction

 Current rates of anthropogenic habitat alteration have raised awareness of a global biodiversity crisis [1]. Declines in species numbers have been reported for a wide variety of taxa [2,3], and extinction rates are expected to increase due to predicted global changes [4]. In addition to direct effects on ecosystem services such as nutrient cycling and primary production [5], species extinctions may lead to the loss of interactions on which other species depend for food, shelter, dispersal and reproduction [6,7]. That is the case for most flowering plants, which depend on animal pollinators for reproduction [8]. While data on pollinator richness and abundance is scarce for many parts of the globe, there is growing concern that pollinators may be declining due to habitat fragmentation, invasion by alien species, use of pesticides and global warming [9–11].

Disruption of pollination by animals may lead to decreased plant productivity and reproductive success [12,13]. Eventually, pollinator extinctions may trigger coextinction cascades in which secondary extinctions of plants cause further extinctions of pollinators and so on [6,7]. Thus, predicted pollinator declines may ultimately lead to the disruption of plant communities, which in turn leads to the collapse of the ecosystem services they maintain [1,5]. Since plant functional diversity is strongly related to ecosystem functioning [14,15], the intensity of the decline in ecosystem functioning will depend partly on how quickly plant functional diversity decreases following plant-pollinator coextinctions. 

In parallel to declines in plant functional diversity, plant extinctions due to the loss of their pollinators imply the loss of the phylogenetic diversity of the plant assemblage [16,17]. Because functional traits are often similar among closely related species [18] functional diversity should be strongly related to phylogenetic diversity, so that the functional and phylogenetic consequences of plant-pollinator coextinctions should be similar. However, some studies have shown that congruence between patterns of functional and phylogenetic diversity does not always occur [19,20]. While simulated coextinctions in mutualistic networks (including pollination networks) may lead to relatively fast declines in phylogenetic diversity [17], the consequences of plant-pollinator coextinctions to plant functional diversity remain to be investigated. If functionally unique plant species are particularly prone to suffer coextinctions, then plant functional diversity should decline rapidly following pollinator losses. On the other hand, if functionally unique plant species are unlikely to suffer secondary extinctions compared to more redundant species, plant functional diversity should be robust to the disruption of pollination services. 

Here, we investigated the loss of plant functional and phylogenetic diversity following pollinator extinctions by simulating coextinctions in empirical, quantitative plant-pollinator networks. We contrasted simulated declines in functional and phylogenetic diversity under a realistic coextinction scenario with declines resulting from optimistic, pessimistic and random scenarios for the loss of functional and phylogenetic diversity. We also looked for possible relationships between species functional and phylogenetic uniqueness and their susceptibility to coextinctions. Finally, we asked whether functionally or phylogenetically similar plant species are at similar risk of being lost in a plant-pollinator coextinction scenario. 

## Materials and Methods

### Compilation of plant-pollinator networks

We performed simulations on empirical, quantitative networks available in the literature or requested directly to authors. A quantitative plant-pollinator network is described by an interaction matrix whose entries *a*_*ij*_ contain the number of times pollinator species *i* was recorded visiting plant species *j*. Thus quantitative interaction networks report a reasonable estimate of the total effect of each mutualistic interaction on the interacting pair of species [21], as well as total interaction frequency for each species. We assume that recorded interactions between plants and insects are actual plant-pollinator interactions; however, we note that many studies do not discriminate between occasional flower visitors and actual pollinators [22]. Interaction frequencies were used here to ascribe relative risks of primary and secondary extinction to species in simulations. Since we were interested in plant functional and phylogenetic diversity, we could only include quantitative networks for which information on both functional traits and phylogeny was available for the plants. Simultaneous availability of both kinds of information is scarce, so that our literature search, resulted in seven networks described in northern and central Europe: Switzerland (Albrecht et al. 2010 [23], data for the 130-year-old site), Scotland (Devoto et al. 2012 [24], old-growth site #30), England (Memmott 1999 [25]; Dicks et al. 2002 [26], Hicking site; both available as supplementary material in [19]), Norway (Hegland et al. 2010 [27], data for 2004), and Germany (Junker et al. 2010 [28], network 1; Weiner et al. 2011 [29], available as supplementary material therein). We refer to each dataset by the name of the respective first author (see Table S1 in Supporting Information for information on network size and connectance).

### Measuring plant functional and phylogenetic diversity

 We estimated plant functional diversity using a suite of traits which capture broad-scale variation in plant ecological strategies: specific leaf area (SLA), plant height and seed mass (LHS scheme, [30]). Each one of these traits represents important trade-offs controlling plant strategies [30] and are related to other important traits [31]. These traits are also associated to plant responses to soil resources, competitive strength, and effects on biogeochemical cycles and productivity [32–34]. Further, such traits are easily measurable, which makes the LHS system broadly used in ecological studies [35–37]. 

 We searched the LEDA database (www.leda-traitbase.org) for data on specific leaf area (SLA), canopy height and seed mass. We included only plant species with data for at least two traits. We removed plants with information for less than two traits from the interaction matrices prior to the simulations and removed any pollinators which had zero interactions after removing such plants (see Appendix S1 in Supporting Information for details on the compilation of trait data and the adjustment of interaction matrices). This resulted in 0–9 plant species being removed (0-38%, median = 8.6%).

 We built a functional dendrogram for the set of plant species in each network using a Euclidean distance matrix and the UPGMA clustering algorithm. We obtained phylogenies for the plant assemblages in each network using a recently published dated phylogeny of European plants [38] encompassing all plant species found in the pollination networks included in this study. For consistency, we removed those plants with insufficient data on functional traits (as defined above and in Appendix S1) from each phylogeny and from subsequent calculations of phylogenetic diversity.

We measured functional and phylogenetic diversity as the sum of branch lengths needed to connect all non-extinct species in the corresponding functional dendrogram or phylogenetic tree: FD and PD, respectively [16,39,40]. Note that FD for a single species assemblage is defined as zero, whereas this is not the case for PD. Each functional dendrogram and phylogenetic tree was built with the complete set of plant species in each network (except for plants with missing traits as defined above) and was not reconstructed during the simulated coextinction sequences.

To estimate the functional and phylogenetic uniqueness of each plant species in each assemblage, we calculated their “originality” from the corresponding functional dendrogram and phylogenetic tree [41]. Originality measures the relative contribution of each species to the overall functional or phylogenetic diversity of the assemblage, such that the values for all species add up to 1. Both functional and phylogenetic originality of each plant species were based on the complete plant assemblage of the network and thus were calculated prior to simulated extinctions. As alternative metrics for functional and phylogenetic diversity, we used “total functional originality” and “total phylogenetic originality”, the sum of functional and phylogenetic originality values across all non-extinct plant species in each network. Since results were qualitatively consistent, we present only the results for FD and PD in the main text. 

### Coextinction model and simulations

To investigate the impact of pollinator extinctions on the functional and phylogenetic diversity of plant communities, we used a simulation approach based on the removal of species from the observed interaction matrices. This is the standard method for estimating the robustness of interaction networks to coextinctions [17,42–44]. However, we acknowledge that this approach may produce biased results for rare species due to undersampling of interactions [22]. 

We developed a stochastic model of coextinctions in mutualistic networks based on network topology and interaction strengths. Contrary to other topological models of coextinctions in ecological networks (e.g. 43), it allows coextinction cascades involving an indefinite number of species to occur following a single episode of primary extinction. We briefly describe the model here in the context of pollination networks and shall discuss its properties in detail elsewhere. A single event of coextinction is modeled as follows. We let *P*_*ij*_ = *R*_*i*_ *d*_*ij*_ be the probability of species *i* suffering extinction following the extinction of a mutualistic partner species *j*, where *d*_*ij*_ is the dependence of species *i* on species *j* and *R*_*i*_ is a constant which reflects the intrinsic reproductive dependence of species *i* on pollination (when *i* is a plant) or its intrinsic dependence on floral resources for food (when *i* is a pollinator). We assumed *R = 1* for all plants and pollinators in this study. Thus, while simulation models usually assume that a species goes extinct only after losing its last mutualistic partner, we relax such assumption in our model. We assume, however, that species cannot establish new mutualistic interactions after the extinction of their original mutualistic partners. Dependence of *i* on *j* is calculated as the number of interactions recorded between that pair of species divided by the total number of interactions of species *i* [45]. Thus an interaction matrix of *a* animals and *p* plants results in two *a* x *p* dependence matrices which describe how much each plant depends on each pollinator and how much each pollinator depends on each plant.

For each pollination network, we simulated coextinction sequences involving primary extinction episodes and possible coextinction cascades which occurred according to the model described above. Each simulated extinction sequence proceeded until all species had become extinct. A simulation step in each sequence involved the primary extinction of a single pollinator species followed by the update of the interaction matrix and possibly by a sequence of associated extinctions. Extinctions were represented in the interaction matrix by setting all entries of a row (pollinator) or column (plant) to zero. At the beginning of each step, a single pollinator species was chosen as a target for primary extinction. All plant species then had a chance of suffering secondary extinction according to the model described above: for each plant species, a value between 0 and 1 was sampled from a uniform distribution, and a species was considered extinct if such value was smaller than the species *P*_*ij*_ value. If any plant species went secondarily extinct, all of the surviving pollinator species in turn had a chance of going extinct themselves, and so on until the sequence was interrupted by no further extinctions occurring. Then we assumed the community reached equilibrium, calculated FD and PD for the set of surviving plant species and moved on to the next primary extinction episode. The algorithm updated the dependence matrix as the extinction sequence moved forward. We quantified the persistence of a plant species in an extinction sequence as the number of primary extinction episodes which occurred before that species was lost. 

Primary extinctions of pollinators at the beginning of each simulation step took place in a realistic scenario in which pollinator species with lower total interaction frequencies had a higher chance of suffering primary extinctions at each step. In pollination networks total interaction frequency tends to be strongly correlated with abundance [46,47], which is in turn a proxy to extinction risk. We assumed that the probability of primary extinction for each pollinator species is proportional to the inverse of its total interaction frequency. We ran 10^4^ coextinction sequences and calculated the average curve describing the decline in FD and PD for each network, as well as the average persistence of each plant species. Each average curve describes the decline in functional or phylogenetic diversity as a function of the proportion of plant species lost. 

In order to provide a framework to interpret declines in plant functional and phylogenetic diversity, we calculated a second set of curves for each network which described the decline in FD and PD when plant species were lost independently of their pollinators and according to three reference scenarios: (1) a best-case scenario in which plants species were removed deterministically in increasing order of originality (functional or phylogenetic, separately); (2) a worst-case scenario in which plants species were removed deterministically in decreasing order of originality; and (3) a random scenario (10^4^ simulations). Best- and worst-case scenarios set boundaries within which any decline in FD and PD should lie. We implemented all simulations in R [48] using package ‘ade4’ to calculate originality [49] and package ‘picante’ to calculate FD and PD [50]. 

### Statistical analyses

We performed Spearman correlation tests to assess whether persistence was associated with functional and phylogenetic originality. To assess the degree to which functionally or phylogenetically similar plant species shared similar risk of suffering coextinction, we performed autocorrelation analyses by calculating Moran’s correlograms for persistence using distance matrices built from the functional dendrograms and phylogenetic trees for each network [51]. Also, because coupled responses of functional and phylogenetic diversity require functional traits to be conserved to some degree along lineages, we quantified phylogenetic signal in the functional originality of species by calculating phylogenetic correlograms for functional originality in each network. We conducted all autocorrelation analyses in PAM v0.9 (Phylogenetic Analysis in Macroecology; [52]).

## Results

**Figure 1:**
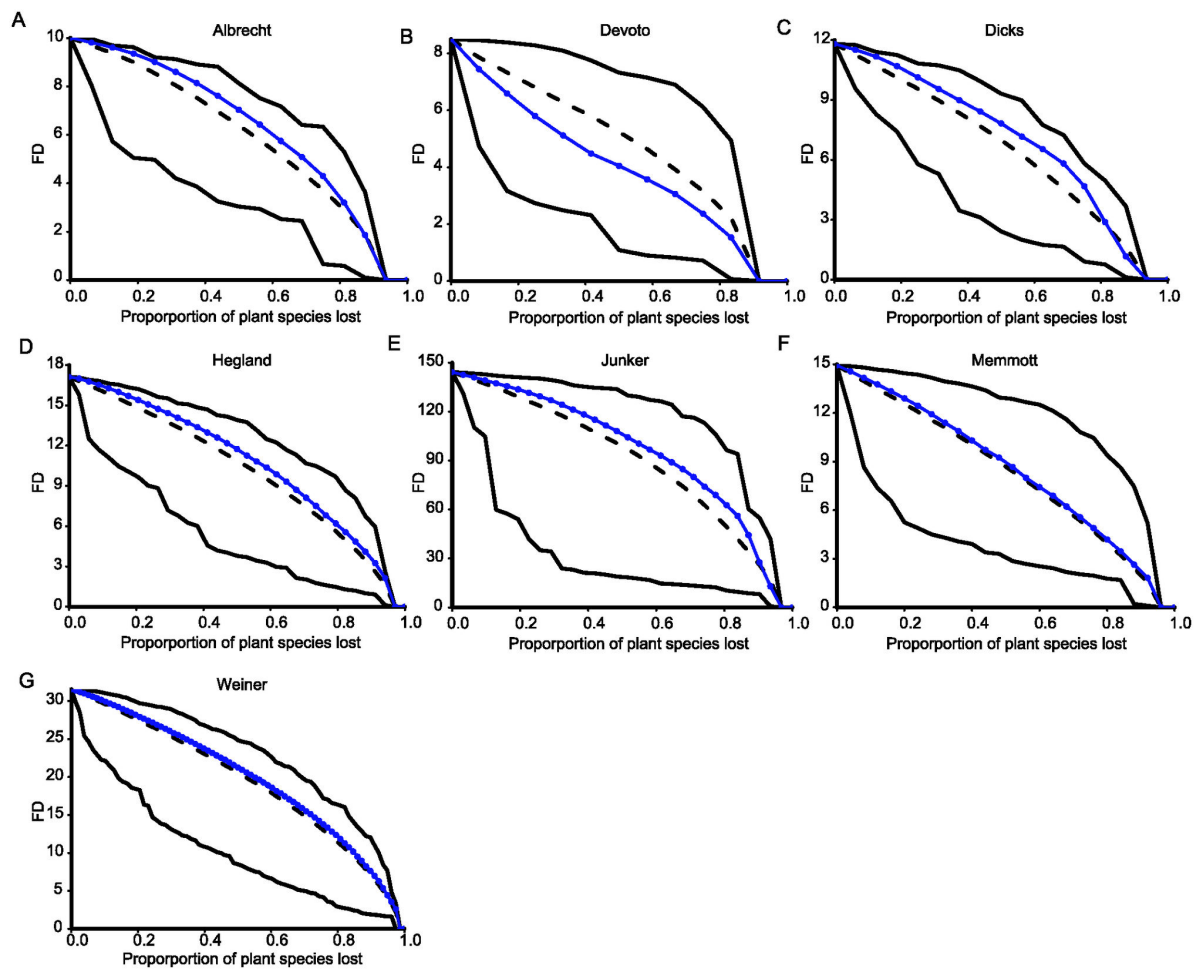
Declines in functional diversity (FD) following simulated plant-pollinator coextinctions in seven pollination networks (A-G). Circles: declines following plant-pollinator coextinctions. Dotted lines: declines following random plant extinctions in the absence of coextinctions. Solid lines above and below the dotted lines represent best- and worst-case scenarios, respectively.

**Figure 2:**
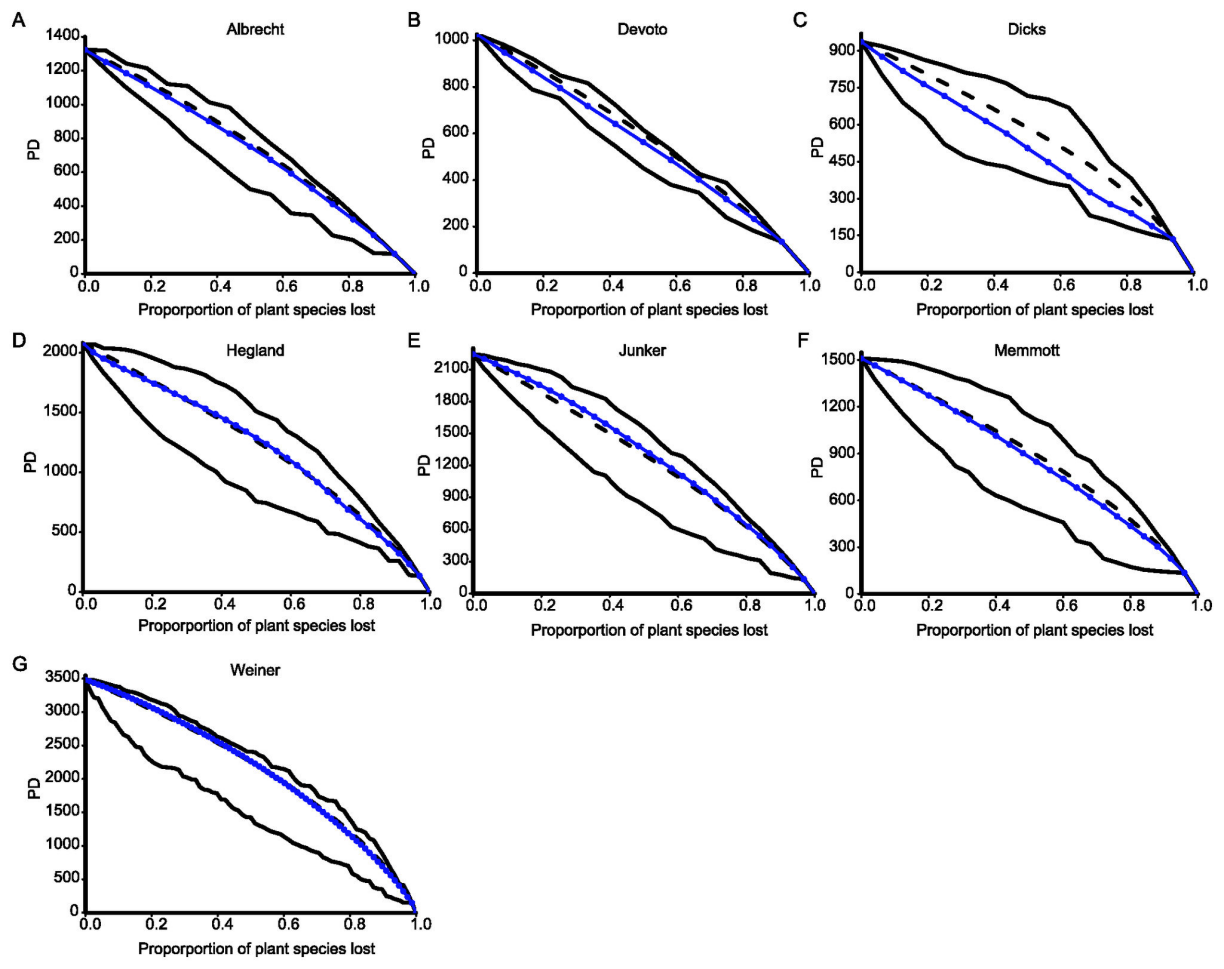
Declines in phylogenetic diversity (PD) following simulated plant-pollinator coextinctions in seven pollination networks (A-G). Circles: declines following plant-pollinator coextinctions. Dotted lines: declines following random plant extinctions in the absence of coextinctions. Solid lines above and below the dotted lines represent best- and worst-case scenarios, respectively.

**Figure 3:**
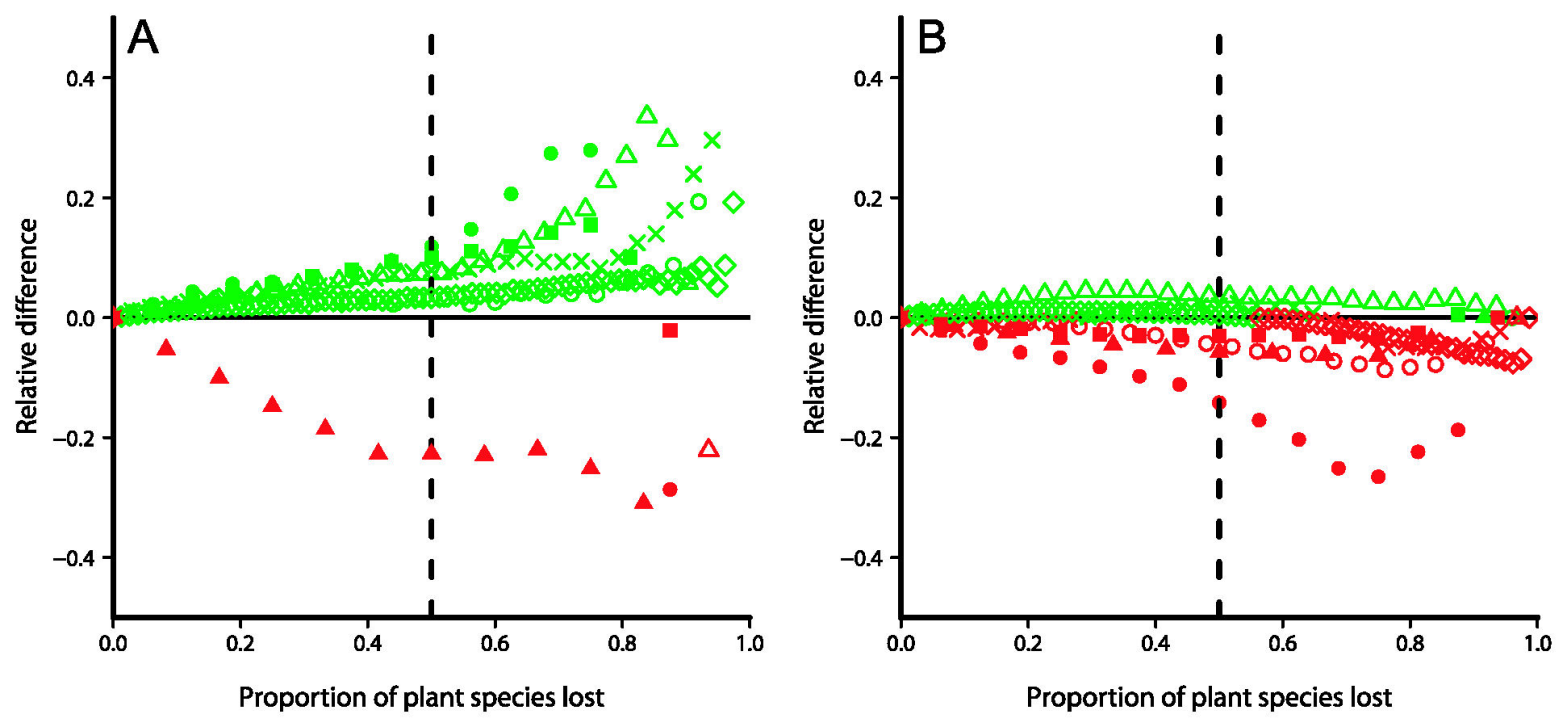
Relative declines in FD and PD. Relative difference between declines in FD (A) and PD (B) under a plant-pollinator coextinction scenario and declines under a random scenario of plant extinctions in the absence of coextinctions. Positive and negative deviations from the random expectation are shown in green and red, respectively. Closed squares = Albrecht, closed triangles = Devoto, closed circles = Dicks, crosses = Hegland, open triangles = Junker, open circles = Memmott, diamonds = Weiner. Vertical dotted line indicates values when 50% of plant species have been lost.

 Declines in FD and PD associated with simulated plant-pollinator coextinctions are shown in Figures 1 and 2 (see also Figures S1 and S2 in Supporting Information for results obtained using total functional and phylogenetic originality, respectively). In six out of seven networks, FD decreased consistently more slowly than expected under a random scenario (Figures 1, 3A). In those cases, the relative extra amount of functional diversity preserved in comparison with the random scenario ranged from 3.1% (Memmott network; Figures 1F, 3A) to 11.9% (Dicks network; Figures 1C, 3A) at the point when 50% of all plant species had been lost. In the Devoto network, however, FD decreased consistently faster than expected under the random scenario (Figure 1B) and was 22.9% smaller than the random expectation at the point when 50% of plant species had been lost (Figure 3A).

 In all networks except Devoto, PD decreased faster than FD when both were compared to their respective random expectation (Figure 3). In four networks (Albrecht, Devoto, Dicks and Memmott), declines in PD were consistently faster than expected under the random scenario of plant extinctions (Figures 2A-C,F; Figure 3B), so that PD was 3.0–14.2 % smaller than the random expectation at the point when 50% of plant species had been lost. Consistently slower-than-random declines in PD occurred in only one network (Junker), so that PD was 3.5% greater than expected under the random scenario following the loss of 50% of plant species (Figures 2E, 3B). The two remaining networks exhibited slower-than-random declines in PD up to the point when about 55% (Weiner; Figure 2G) and 67% (Hegland; Figure 2D) of plant species had suffered coextinction, and negative deviations from the random curve after that point. 

**Figure 4:**
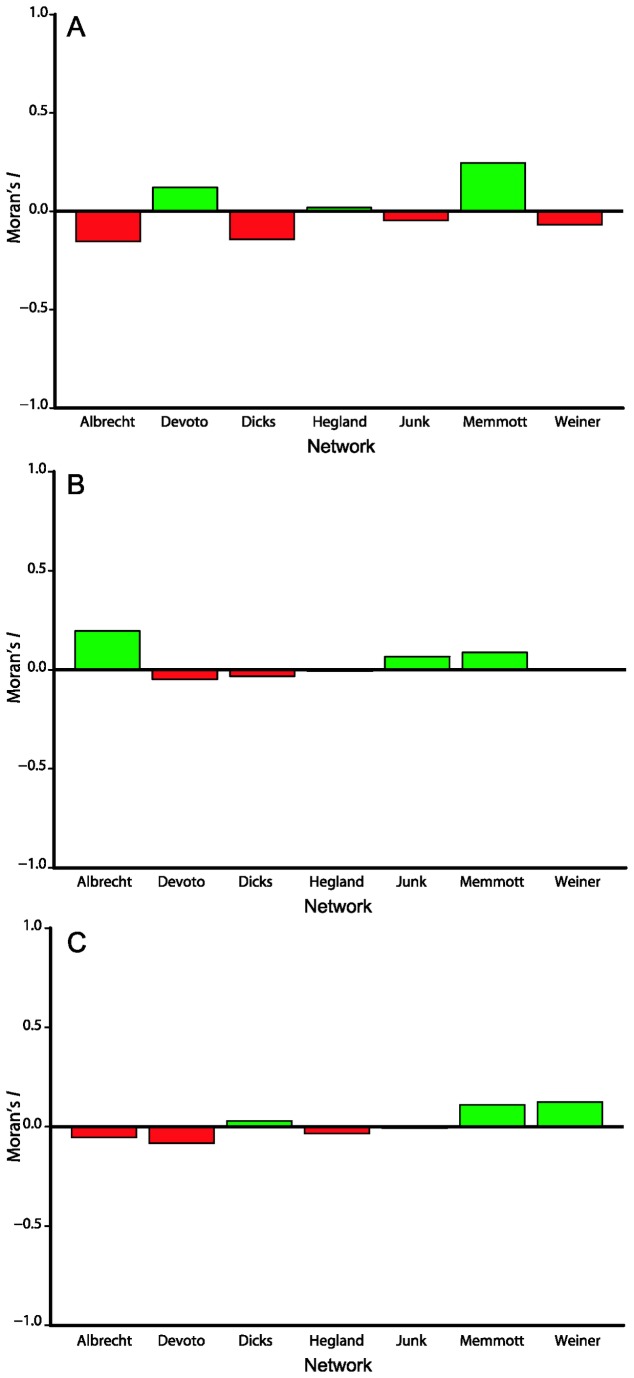
Autocorrelation analyses. (A) Autocorrelation in persistence among plant species close to each other in the functional dendrogram. (B) Autocorrelation in persistence among phylogenetically close plant species. (C) Autocorrelation in functional originality among phylogenetically close plant species. Moran’s I values were calculated with respect to the first distance class in the correlogram.

 Species functional originality showed strongly asymmetric frequency distributions in all pollination networks, with most plant species having very low functional originality and single species accounting for 17.0–41.1% of the total (Figure S3). We found no correlation between species persistence and species functional originality in any of the networks (Figure S3; Table S2). Phylogenetic originality was more evenly distributed across plant species in pollination networks, with the single most phylogenetically original species in each network accounting for 10.3–24.5% of total phylogenetic originality. We also found no correlation between species phylogenetic originality and species persistence in 6 out of 7 networks (Figure S4; Table S1). In the Devoto network, species with high phylogenetic originality had lower persistence and thus higher risk of suffering coextinction (rs = -0.643; p = 0.028). Overall, functionally similar or phylogenetically close plant species had no tendency to have similar persistence to coextinctions (Figure 4A-B, Table S3). Also, phylogenetically close plant species had no tendency to have similar functional originality (Figure 4C, Table S3), which suggests no overall phylogenetic signal in the set of functional traits used.

## Discussion

 Overall, coextinction trajectories led to slower declines in plant functional diversity than expected under a scenario in which plant functional diversity was lost at random. In contrast, phylogenetic diversity decreased faster than functional diversity in all networks except one, and faster-than-random declines in phylogenetic diversity occurred in four of them. Thus, our results show that the loss of plant functional diversity is not necessarily coupled with the decline in plant phylogenetic diversity following the loss of their pollinators. While the absence of phylogenetic signal in functional traits would by itself suggest that functional diversity might not track phylogenetic diversity in its faster decline, our results show that declines in functional diversity may actually deviate from the random expectation in the opposite direction. Thus, we confirm the previous finding that phylogenetic diversity is often lost rapidly following coextinctions in mutualistic networks [17], and we also show that plant functional diversity is relatively robust to pollinator extinctions despite a relatively faster underlying loss of plant evolutionary history. 

 The possibility of uncoupled functional and phylogenetic consequences of plant-pollinator coextinctions highlights the importance of taking functional diversity explicitly into account in ecological studies and when planning for the conservation of species and their interactions, instead of simply taking phylogenetic diversity as a proxy. Previous work has found mismatches between functional and phylogenetic diversity in their spatial distribution [19,20] and in the extent to which they are represented by indicator groups in a conservation context [53]. Our results further suggest that, even when functional and phylogenetic diversity do exhibit congruence in space, such local congruence may eventually be lost due to uncoupled responses to species coextinctions. Consequently, because functional rather than phylogenetic diversity is an ultimate driver of ecosystem functioning [54,55], predicting declines in ecosystem functioning from declines in phylogenetic diversity may lead to erroneous conclusions if both dimensions of biodiversity respond in different ways. 

 Non-random loss of functional diversity and ecosystem function in plant communities under non-random extinction scenarios has been demonstrated before in computer simulations [39,56] and experimentally [57,58]. Those studies have explored different classes of realistic extinction scenarios, such as due to climate change or different management and harvesting strategies of plant communities, and then simulated plant extinctions based on traits likely to be associated with extinction risk in each scenario [39,56,57] or on observed nested patterns of species occurrence [58]. We propose the modeling of mutualistic coextinctions, previously used to study the loss of plant phylogenetic diversity [17], as an additional strategy for building realistic scenarios aimed at exploring the functional consequences of plant extinctions. Extinction risk in this type of scenario is linked to the architecture of species interactions instead of being directly linked to morphological or physiological traits, and realism can be achieved by considering the empirical pattern of interactions described in mutualistic networks. 

 Although non-random declines in functional and phylogenetic diversity occurred, we found no relationship between the functional and phylogenetic uniqueness of plant species and their risk of suffering coextinction, nor did we find any tendency for functionally of phylogenetically similar plant species to have similar coextinction risk. It is possible that non-random declines result from one or a few plant species in each network contributing disproportionately to the functional and phylogenetic diversity of the plant assemblage. Because the loss of those highly unique species is associated with the loss of large amounts of functional and phylogenetic diversity, overall declines in those variables may be effectively determined by the particular persistence of those species. For example, in the only network which exhibited a faster-than-random decline in functional diversity under the coextinction scenario (Devoto network), two of the three most sensitive species accounted for more than 60% of total functional originality. In contrast, the single most persistent species accounted for about 40% of total originality in the Junker network, in which functional diversity decreased more slowly than the random expectation. Since the overall pattern seems to be slower-than-random declines in functional diversity, our results suggest that plant species which contribute disproportionately to functional diversity are relatively well-protected against the loss of pollinators, even if no general relationship can be found among the whole plant assemblage. On the other hand, since phylogenetic diversity decreased faster than functional diversity, and often faster than expected under the random scenario, it appears that highly phylogenetically unique plant species are often sensitive to the loss of their pollinators.

 While we provide a first assessment of the functional consequences of coextinctions in mutualistic networks, the effect of predator-prey coextinctions on the functional diversity of food webs has been investigated before. It has been shown that simulated coextinctions lead to greater-than-random loss of total trophic diversity in model and natural food webs [59]. In contrast, while we did find non-random declines in the functional diversity of pollination networks, they were mainly in the opposite direction. Also, we found no association between functional uniqueness and probability of coextinction among plant species, while in food webs more functionally unique species seem to have higher interaction frequencies [60] and higher probability of suffering secondary extinctions [59]. While such food web studies focused on traits involved in the realization of the predator-prey interactions, we estimated functional diversity by considering non-reproductive traits related to broad variation in plant strategies and related to ecosystem functions such as nutrient cycling and productivity. Thus, it is possible that functional and phylogenetic diversity show coupled responses to plant-pollinator coextinctions if functional diversity is estimated using reproductive (e.g. floral, phenological) traits linked to pollination interactions and to the structure of pollination networks. 

 It remains to be investigated whether similar results are to be found when considering plant-pollinator communities from different regions of the globe. Different patterns of decline in functional diversity might arise since, for example, structural properties of mutualistic networks have been shown to vary along latitudinal and altitudinal gradients [61,62]. Whether the degree of uncoupling between functional and phylogenetic diversity varies geographically and can be predicted on the basis of network properties is also open to investigation. Also, we do not know whether coextinctions due to the loss of other kinds of mutualistic partners would produce similar impacts on plant functional diversity. Seed dispersers such as birds, for example, are endangered due to threats similar to those faced by pollinators [63], and the architecture of seed dispersal networks is generally similar to that of pollination networks [47]. Finally, pollinator behavior may influence the persistence of plant species since pollinators may switch to new plant species following declines in the abundance of their original partners [64]. If the probability of a plant species being visited by additional pollinator species is correlated to its functional and phylogenetic originality, the effect of plant-pollinator coextinctions on plant functional and phylogenetic diversity may differ from what is suggested by our results. 

 In conclusion, our results point towards distinct consequences of mutualistic coextinctions to the functional and phylogenetic diversity of plant assemblages. Investigating the causes of such uncoupling, in terms of network structure, and its implications, in terms of predicting community and ecosystem responses to environmental change, can improve our understanding of the consequences of species extinctions.

## Supporting Information

Appendix S1 **Compilation of trait data and adjustment of interaction matrices.**
(PDF)

Table S1 **Summary of network properties for the seven plant-pollinator networks used in the simulations.**
(PDF)

Table S2 **Spearman correlation tests between plant functional/phylogenetic originality and persistence to coextinctions following the loss of pollinators.**
(PDF)

Table S3 **Results from autocorrelation analyses.**
(PDF)

Figure S1 **Declines in total functional originality, following simulated plant-pollinator coextinctions in seven pollination networks (**A**-**G**).** Circles: declines following plant-pollinator coextinctions. Dotted lines: declines following random plant extinctions in the absence of coextinctions. Solid lines above and below the dotted lines represent best- and worst-case scenarios, respectively.
(PDF)

Figure S2 **Declines in total phylogenetic originality, following simulated plant-pollinator coextinctions in seven pollination networks (**A**-**G**).** Circles: declines following plant-pollinator coextinctions. Dotted lines: declines following random plant extinctions in the absence of coextinctions. Solid lines above and below the dotted lines represent best- and worst-case scenarios, respectively.
(PDF)

Figure S3 **Functional originality and ranked persistence values for plant species in the seven plant-pollinator networks (**A**-**G**).**
(PDF)

Figure S4 **Phylogenetic originality and ranked persistence values for plant species in the seven plant-pollinator networks (**A**-**G**).**
(PDF)
