# Supplementary material for: Plant-Pollinator Coextinctions and the Loss of Plant Functional and Phylogenetic Diversity
Source: PLoS One. 2013 Nov 29;8(11):e81242. doi: 10.1371/journal.pone.0081242 (PMC3843674; doi:10.1371/journal.pone.0081242)
Supplement: Appendix S1 — Compilation of trait data and adjustment of interaction matrices. (PDF) [file pone.0081242.s001.pdf]

Appendix S1 – Compilation of trait data and adjustment of interaction matrices

We searched the LEDA database (www.leda-traitbase.org) for information on specific leaf area (SLA), canopy height and seed mass for the plant species in all seven pollination networks. We obtained raw data from the database between March and August 2012. We pooled pre-aggregated entries for each species according to the following criteria: for SLA, we averaged all entries coming from measurements performed on adult individuals following rehydration (for plant species in all networks except Albrecht), in accordance with a protocol proposed by Cornelissen et al. (2003). For plant species in the Albrecht network, we averaged all entries performed on adult individuals without prior rehydration, since measurements following rehydration were missing for many species. For canopy height, we averaged all entries for each species. For seed mass, we averaged all entries for each species, except for entries coming from measurements which reported the inclusion of seed appendages. Prior to coextinction simulations, we removed plant species with information for less than two functional traits from their respective networks, after checking whether data were available under species synonyms (checked at www.theplantlist.org). This resulted in nine plant species being removed from the Albrecht network (*Cardamine resedifolia*, *Sempervivum montanum*, *Trifolium pallescens*, *Laserpitium halleri*, *Campanula barbata*, *Galium anisophyllon*, *Achillea erba-rota* ssp. *moschata*, *Taraxacum* sp. and *Hieracium stacitifolium*), two from Devoto ("unidentified Gramineae" and *Plantago* sp.), one from Dicks (*Taraxacum officinale*), four from Hegland (*Alchemilla* sp., *Euphrasia stricta*, *Rosa* sp. and *Valeriana sambuccifolia*), three from Junker (*Erigeron anuus*, "*Rosa* spec. 1" and *Capsicum pubescens*) and three from Weiner (*Medicago varia*, *Orobranche* sp. and *Taraxacum officinale*). We also removed plants with zero interactions in the

original matrices provided by M. Albrecht (Albrecht network; *Rumex scutatus*, *Cardamine resedifolia*, *Rhododendron ferrugineum*, *Pyrola minor*, *Sempervivum montanum*, *Sempervivum arachnoideum*, *Trifolium pratense*, *Trifolium pallescens*, *Melampyrum silvaticum*, *Campanula barbata*, *Galium anisophyllon* and *Leontodon helveticus*) and obtained from Weiner et al. (2011) (Weiner network; *Myosotris sylvatica*). We also removed any pollinators which had zero interactions following the removal of plants: one from Devoto (pollinator #9) five from Albrecht (pollinators #15, #18, #20, #23, #29); three from Hegland (*Conops quadrifasciatus*, *Eristalis pertinax*, *Opomyza petrei*,); 13 from Junker (Tachinidae spp. 1 & 6, Vespidae spp. 3 & 4, Chrysanelidae sp.1, Curculionidae sp.2, Dermaptera sp. 3, Heteroptera spp. 17, 22 & 30, Ichneumonidae sp. 2, *Melieria crassipennis* and Thomisoidea sp. 1) and two from Weiner (*Megachile alpicola* and *Mechachile nigriventris*).

REFERENCES

Cornelissen, J.H.C., Lavorel, S., Garnier, E., Díaz, S., Buchmann, N., Gurvich, D.E., et al. (2003). A handbook of protocols for standardised and easy measurement of plant functional traits worldwide. *Australian Journal of Botany*, 51, 335–380.

Weiner, C.N., Werner, M., Linsenmair, K.E. & Blüthgen, N. (2011). Land use intensity in grasslands : Changes in biodiversity , species composition and specialisation in flower visitor networks. *Basic and Applied Ecology*, 12, 292–299.
