# Supplementary material for: Plant-Pollinator Coextinctions and the Loss of Plant Functional and Phylogenetic Diversity
Source: PLoS One. 2013 Nov 29;8(11):e81242. doi: 10.1371/journal.pone.0081242 (PMC3843674; doi:10.1371/journal.pone.0081242)
Supplement: Table S1 — Summary of network properties for the seven plant-pollinator networks used in the simulations. (PDF) [file pone.0081242.s002.pdf]

Table S1. **Summary of network properties for the seven plant-pollinator networks used in the simulations**.

Network	S	Spol	Spla	Spol / Spla	C
Albrecht [1]	40	24	16	1.500	0.122
Devoto [2]	38	26	12	2.167	0.157
Dicks [3]	77	61	16	3.813	0.149
Hegland [4]	176	142	34	4.176	0.091
Junker [5]	181	150	31	4.839	0.059
Memmott [6]	104	79	25	3.160	0.151
Weiner [7]	594	516	78	6.615	0.045

S = species richness; S_{pol} = Number of pollinator species; S_{pla} = Number of plant species; C = connectance

References

1. Albrecht M, Riesen M, Schmid B (2010) Plant-pollinator network assembly along the chronosequence of a glacier foreland. Oikos 119: 1610–1624.

2. Devoto M, Bailey S, Craze P, Memmott J (2012) Understanding and planning ecological restoration of plant-pollinator networks. Ecology Letters: 319–328.

3. Dicks L V, Corbet SA, Pywell RF (2002) Compartmentalization in plant – insect flower visitor webs. Journal of Animal Ecology 71: 32–43.

4. Hegland SJ, Dunne J, Nielsen A, Memmott J (2010) How to monitor ecological communities cost-efficiently : The example of plant – pollinator networks.

5. Junker RR, Höcherl N, Blüthgen N (2010) Responses to olfactory signals reflect network structure of flower-visitor interactions. Journal of Animal Ecology 79: 818–823.

6. Memmott J (1999) The structure of a plant-pollinator food web. Ecology Letters 2: 276–280.

7. Weiner CN, Werner M, Linsenmair KE, Blüthgen N (2011) Land use intensity in grasslands : Changes in biodiversity , species composition and specialisation in flower visitor networks. Basic and Applied Ecology 12: 292–299.
