# Supplementary material for: Plant-Pollinator Coextinctions and the Loss of Plant Functional and Phylogenetic Diversity
Source: PLoS One. 2013 Nov 29;8(11):e81242. doi: 10.1371/journal.pone.0081242 (PMC3843674; doi:10.1371/journal.pone.0081242)
Supplement: Table S2 — Spearman correlation tests between plant functional/phylogenetic originality and persistence to coextinctions following the loss of pollinators. (PDF) [file pone.0081242.s003.pdf]

Table S2. **Spearman correlation tests between plant functional/phylogenetic originality and persistence to coextinctions following the loss of pollinators**.

	Functional originality		Phylogenetic originality	
**Network**	**ρ**	**p**	**ρ**	**P**
Albrecht [1]	0.412	0.113	-0.358	0.173
Devoto [2]	-0.376	0.228	**-0.643**	**0.028**
Dicks [3]	0.424	0.102	-0.379	0.148
Hegland [4]	0.314	0.070	0.174	0.325
Junker [5]	0.162	0.383	0.239	0.194
Memmott [6]	0.176	0.399	-0.143	0.495
Weiner [7]	0.197	0.083	-0.020	0.841

References

1. Albrecht M, Riesen M, Schmid B (2010) Plant-pollinator network assembly along the chronosequence of a glacier foreland. Oikos 119: 1610–1624.

2. Devoto M, Bailey S, Craze P, Memmott J (2012) Understanding and planning ecological restoration of plant-pollinator networks. Ecology Letters: 319–328.

3. Dicks L V, Corbet SA, Pywell RF (2002) Compartmentalization in plant – insect flower visitor webs. Journal of Animal Ecology 71: 32–43.

4. Hegland SJ, Dunne J, Nielsen A, Memmott J (2010) How to monitor ecological communities cost-efficiently : The example of plant – pollinator networks.

5. Junker RR, Höcherl N, Blüthgen N (2010) Responses to olfactory signals reflect network structure of flower-visitor interactions. Journal of Animal Ecology 79: 818–823.

6. Memmott J (1999) The structure of a plant-pollinator food web. Ecology Letters 2: 276–280.

7. Weiner CN, Werner M, Linsenmair KE, Blüthgen N (2011) Land use intensity in grasslands : Changes in biodiversity , species composition and specialisation in flower visitor networks. Basic and Applied Ecology 12: 292–299.
