# Supplementary material for: Plant-Pollinator Coextinctions and the Loss of Plant Functional and Phylogenetic Diversity
Source: PLoS One. 2013 Nov 29;8(11):e81242. doi: 10.1371/journal.pone.0081242 (PMC3843674; doi:10.1371/journal.pone.0081242)
Supplement: Table S3 — Results from autocorrelation analyses. (PDF) [file pone.0081242.s004.pdf]

Table S3. **Results from autocorrelation analyses**.

	Autocorrelation in persistence among functionally close species				Autocorrelation in persistence among phylogenetically close plant species				Autocorrelation in functional originality among phylogenetically close plant species			
**Network**	**I**	**Imax**	**n**	**Class center**	**I**	**Imax**	**n**	**Class center**	**I**	**Imax**	**n**	**Class center**
Albrecht [1]	-0.153	0.561	52	0.665	0.195	1.16	50	106.1	-0.054	0.694	50	106.1
Devoto [2]	0.121	0.73	34	0.377	-0.048	0.765	34	135.6	-0.082	0.703	34	135.6
Dicks [3]	-0.142	0.784	48	0.604	-0.032	0.83	48	48.5	0.029	1.576	48	48.5
Hegland [4]	0.018	0.472	188	0.484	-0.006	0.254	224	101.7	-0.034	0.564	186	97.7
Junker [5]	-0.046	0.553	154	2.348	0.066	0.577	188	113.1	-0.007	0.216	154	113.1
Memmott [6]	0.245	1.166	102	0.493	0.087	0.722	146	98.65	0.11	0.67	102	98.65
Weiner [7]	-0.068	0.536	774	0.533	-0.001	0.245	1062	95.2	0.124	0.646	760	90.55

References

1. Albrecht M, Riesen M, Schmid B (2010) Plant-pollinator network assembly along the chronosequence of a glacier foreland. Oikos 119: 1610–1624.

2. Devoto M, Bailey S, Craze P, Memmott J (2012) Understanding and planning ecological restoration of plant-pollinator networks. Ecology Letters: 319–328.

3. Dicks L V, Corbet SA, Pywell RF (2002) Compartmentalization in plant – insect flower visitor webs. Journal of Animal Ecology 71: 32–43.

4. Hegland SJ, Dunne J, Nielsen A, Memmott J (2010) How to monitor ecological communities cost-efficiently : The example of plant – pollinator networks.

5. Junker RR, Höcherl N, Blüthgen N (2010) Responses to olfactory signals reflect network structure of flower-visitor interactions. Journal of Animal Ecology 79: 818–823.

6. Memmott J (1999) The structure of a plant-pollinator food web. Ecology Letters 2: 276–280.

7. Weiner CN, Werner M, Linsenmair KE, Blüthgen N (2011) Land use intensity in grasslands : Changes in biodiversity , species composition and specialisation in flower visitor networks. Basic and Applied Ecology 12: 292–299.
