# Supplementary figures and images for: Plant-Pollinator Coextinctions and the Loss of Plant Functional and Phylogenetic Diversity
Source: PLoS One. 2013 Nov 29;8(11):e81242. doi: 10.1371/journal.pone.0081242 (PMC3843674; doi:10.1371/journal.pone.0081242)

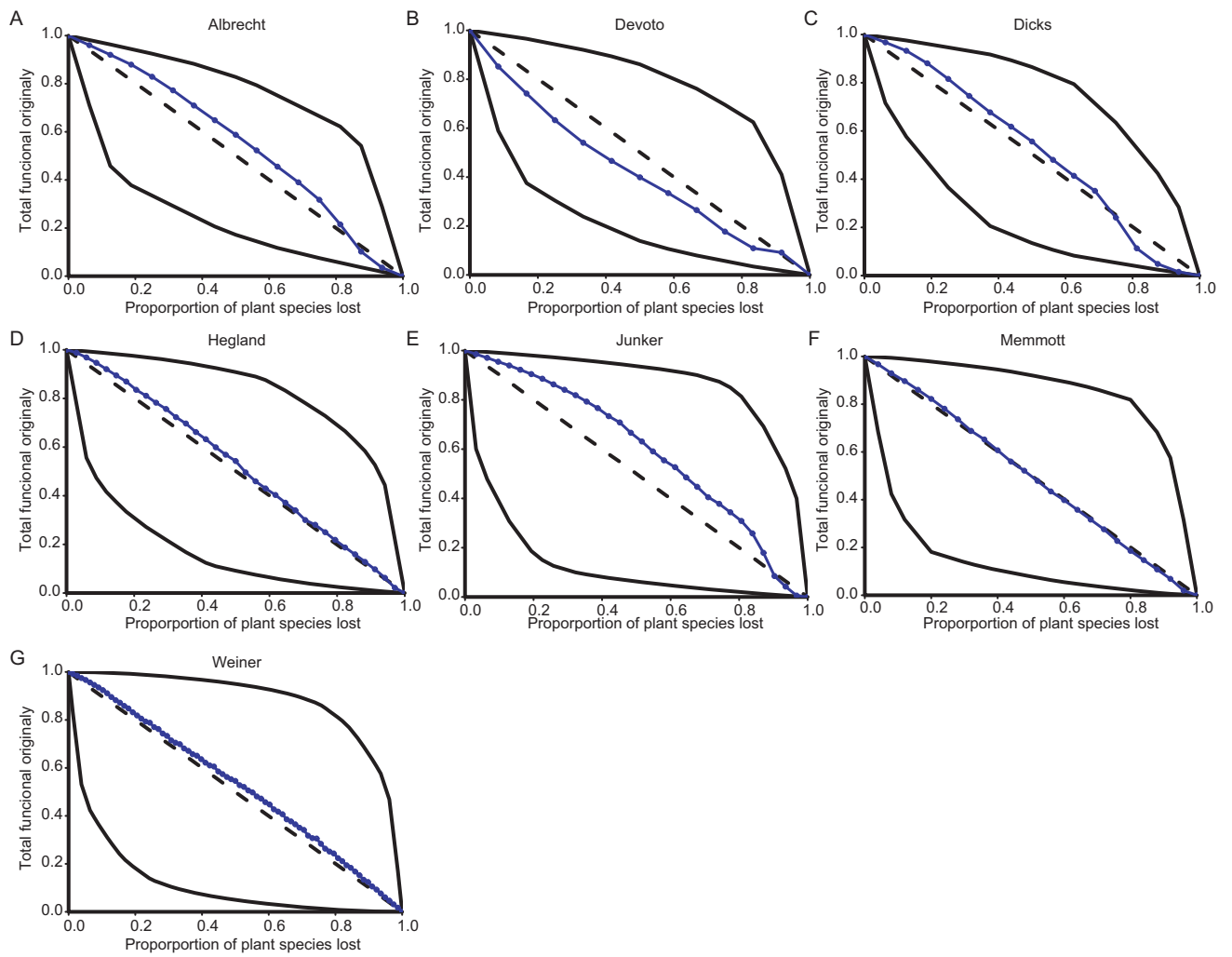

Supplement: Figure S1 — Declines in total functional originality, following simulated plant-pollinator coextinctions in seven pollination networks (A-G). Circles: declines following plant-pollinator coextinctions. Dotted lines: declines following random plant extinctions in the absence of coextinctions. Solid lines above and below the dotted lines represent best- and worst-case scenarios, respectively. (PDF) [file pone.0081242.s005.pdf]

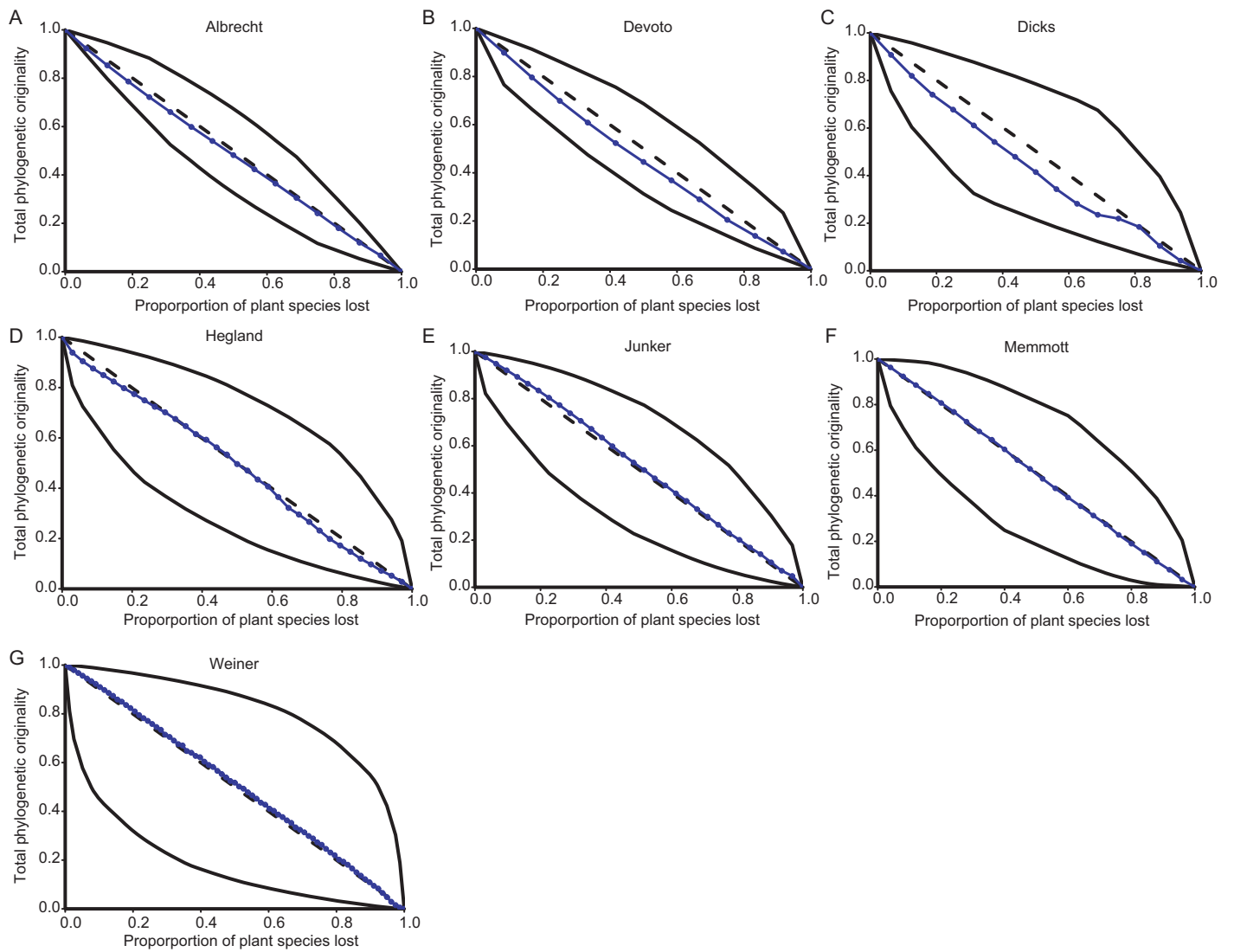

Supplement: Figure S2 — Declines in total phylogenetic originality, following simulated plant-pollinator coextinctions in seven pollination networks (A-G). Circles: declines following plant-pollinator coextinctions. Dotted lines: declines following random plant extinctions in the absence of coextinctions. Solid lines above and below the dotted lines represent best- and worst-case scenarios, respectively. (PDF) [file pone.0081242.s006.pdf]

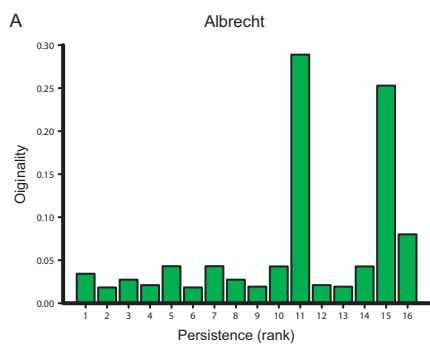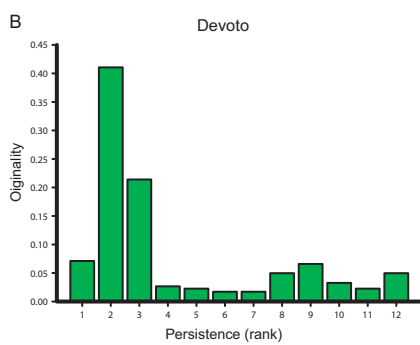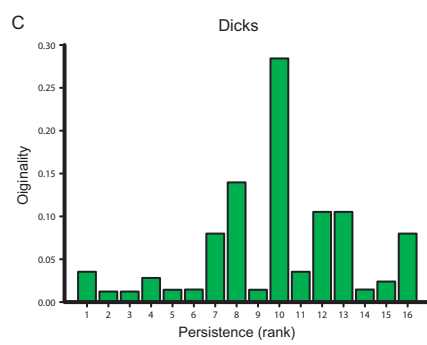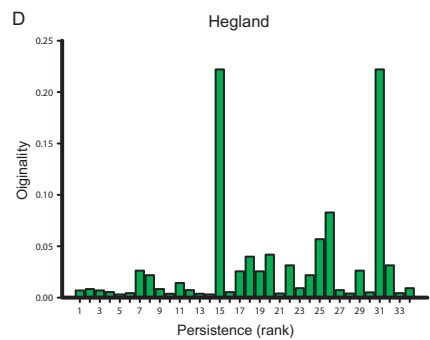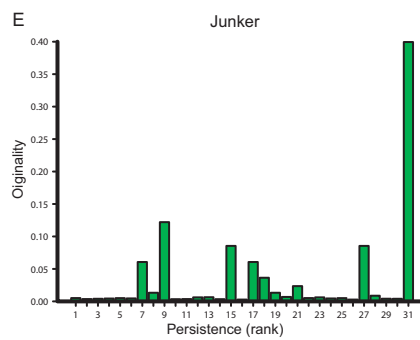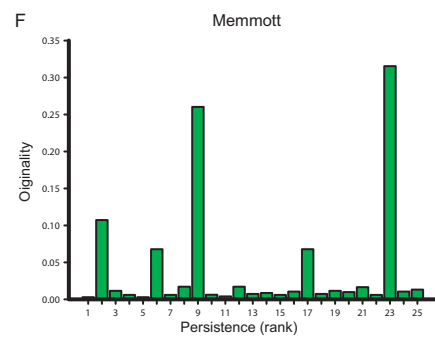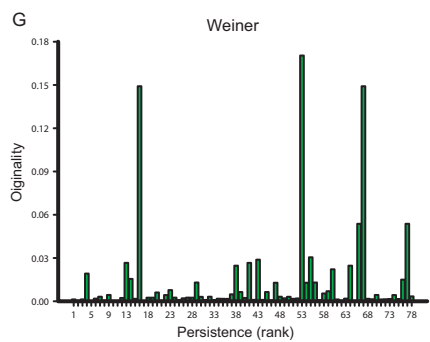

Supplement: Figure S3 — Functional originality and ranked persistence values for plant species in the seven plant-pollinator networks (A-G). (PDF) [file pone.0081242.s007.pdf]

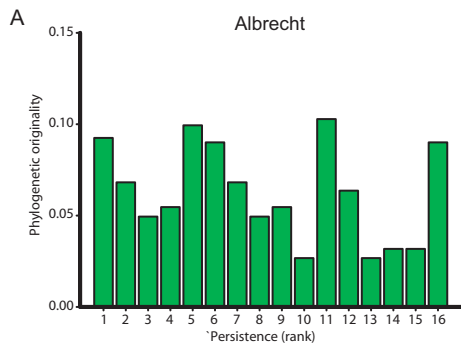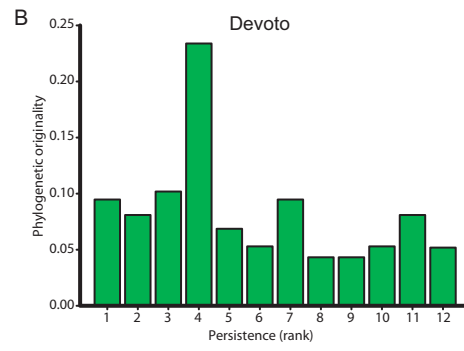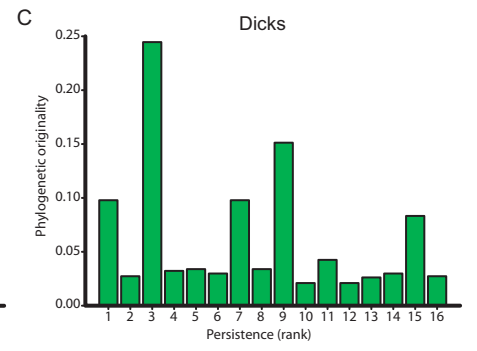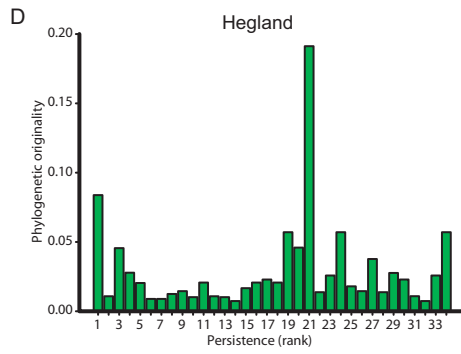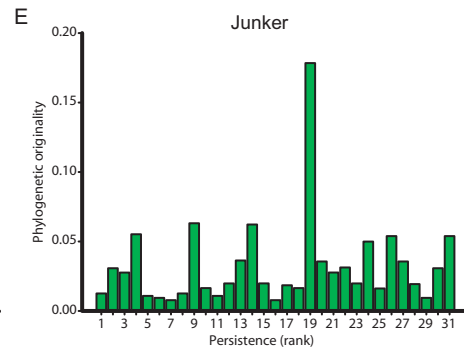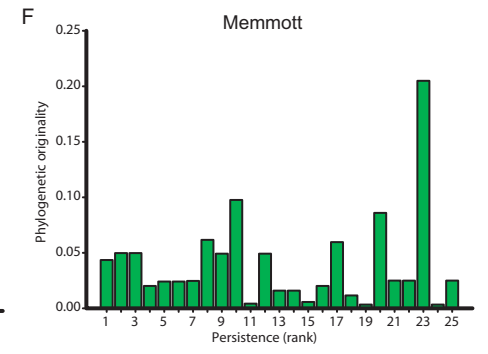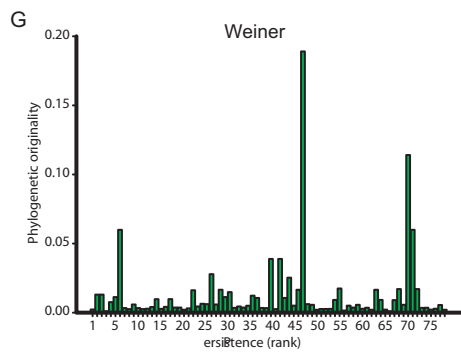

Supplement: Figure S4 — Phylogenetic originality and ranked persistence values for plant species in the seven plant-pollinator networks (A-G). (PDF) [file pone.0081242.s008.pdf]
